# Organic extracts from *Indigofera suffruticosa* leaves have antimicrobial and synergic actions with erythromycin against *Staphylococcus aureus*

**DOI:** 10.3389/fmicb.2015.00013

**Published:** 2015-02-02

**Authors:** Ana Thereza Bezerra dos Santos, Tiago Ferreira da Silva Araújo, Luis Cláudio Nascimento da Silva, Cleideana Bezerra da Silva, Antonio Fernando Morais de Oliveira, Janete Magali Araújo, Maria Tereza dos Santos Correia, Vera Lúcia de Menezes Lima

**Affiliations:** ^1^Laboratório de Química e Metabolismo de Lipídios e Lipoproteínas, Departamento de Bioquímica, Centro de Ciências Biológicas, Universidade Federal de PernambucoRecife, Brazil; ^2^Laboratório de Glicoproteínas, Departamento de Bioquímica, Centro de Ciências Biológicas, Universidade Federal de PernambucoRecife, Brazil; ^3^Laboratório de Ecologia Aplicada e Fitoquímica, Departamento de Botânica, Centro de Ciências Biológicas, Universidade Federal de PernambucoRecife, Brazil; ^4^Laboratório de Genética de Microrganismos, Departamento de Antibióticos, Centro de Ciências Biológicas, Universidade Federal de PernambucoRecife, Brazil

**Keywords:** plant extracts, antibacterial agents, macrolide antibiotic, *S. aureus*

## Abstract

A characteristic feature of *Staphylococcus aureus* is its ability to acquire resistance to antimicrobial agents. There is a need, therefore, for new approaches to combat this pathogen; for example, employing a combination of plant-derived products and antibiotics to overcome bacterial resistance. *Indigofera suffruticosa* is a plant popularly used to treat infections and has verified antimicrobial action. Here, we investigate the antimicrobial activity of different extracts from *I. suffruticosa* against *S. aureus* and their synergistic effects with erythromycin. Leaves of *I. suffruticosa* were extracted sequentially using diethyl ether, chloroform and acetone and the antimicrobial activity of each extract then tested against nine clinical isolates of *S. aureus*. Minimal inhibitory concentration (MIC) and minimal bactericidal concentration (MBC) were determined by microdilution tests, while the fractional inhibitory concentration (FIC) was assessed by checkerboard assay. All organic solvent extracts showed antimicrobial activity against *S. aureus* strains. The acetone extract was the most potent inhibitor of *S. aureus* (MIC and MBC of 0.78 and 3.12 mg/mL), followed by the chloroform extract (MIC and MBC of 3.12 and 6.25 mg/mL). Furthermore, acetone or chloroform extracts of *I. suffruticosa* enhanced the activity of erythromycin against *S. aureus* (FIC ≤ 0.5). We conclude that organic extracts from leaves of *I. suffruticosa*, alone or combined with erythromycin, are promising natural products for the development of new anti-*S. aureus* formulations.

## Introduction

Patients in hospital intensive care units are at risk of acquiring nosocomial infections due to the use of invasive devices and/ or extended hospital stay (Streit et al., 2004). Long-term hospitalization may further complicate patient health by exposure to various antimicrobial agents. Additionally, the indiscriminate use of antibiotics in treating infections promotes bacterial evolution and emergence of resistance strains (Palmer and Kishony, 2013; Tavares et al., 2013). *Staphylococcus aureus* is an important pathogen associated with nosocomial human infections, and this microorganism has successfully evolved numerous strategies to resist different antibiotics (Coutinho et al., 2009; Chung et al., 2011). Such increases in antibiotic resistant *S. aureus* strains drives research discovery of new antimicrobial agents and the development of alternative therapeutic strategies. These include plant extracts, which have considerable antimicrobial potential (Leite et al., 2006; da Silva et al., 2013; Zakavi et al., 2013).

Medicinal plants are important health and economic components used by many cultures for thousands of years (Agra et al., 2008; Silva et al., 2012). According to the World Health Organization approximately 80% of the global population uses medicinal plants or herbal medicine for primary health care (Pereira et al., 2012). Brazil has the highest plant diversity of any country and represents 20% of biodiversity in the world. *Indigofera suffruticosa* Mill (Fabaceae, Papilionidae) is a plant originally from Antilles and Central America, popularly known as “anileira” or “anil,” and was introduced into Brazil for the extraction of indigo, a blue dye blue (Indigo Blue) widely used by the textile industry. Although some toxic effects are reported for this plant, such as hemolytic anemia and hemoglobinuria in cattle and guinea pig (Salvador et al., 2011), it has been used in traditional medicine both externally and internally (Barros and Teixeira, 2008). Moreover, pharmacological effects of *I. suffruticosa* have been confirmed scientifically, such as anti-inflammatory (Chen et al., 2013a), anticonvulant (Almeida et al., 2013) and wound healing (Luiz-Ferreira et al., 2011) (Table 1). Previous work by our group has shown that aqueous infusions of *I. suffruticosa* leaves have inhibitory activity against *S. aureus* and dermatophyte strains (fungi) (Leite et al., 2006), though their action against clinical isolates and synergic potential have yet to be studied.

**Table 1:** **Pharmacological potential of *Indigofera suffruticosa***.

**Scientific account**	**Related compounds**
Gastroprotective agent acute ulcer stimulating prostaglandin, mucus and HSP70. (Luiz-Ferreira et al., 2011)	Ethyl acetate fraction from methanolic extract showed the best action and the authors highlighted the role of role of flavonoids and alkaloids presents in AcF as active compounds
*In vivo* action against *Pediculosis capitis* (García Calixto et al., 2011)	An effective treatment using 5% *I. suffruticosa* Mill tincture was reported in a patient infested with *Pediculosis capitis*
Immunostimulatory and antitumoral actvities *in vitro* (Lopes et al., 2011)	This study evaluated the action of both alkaloid fraction and pure indigo. Indigo showed high activity which suggest that it is the major active principle in *I. suffruticosa*
Antimycobacterial (Carli et al., 2010)	These authors did not isolate or detected any compounds. The methanolic extract showed better activity than dichloromethane
Anticonvulsant effect (Almeida et al., 2013)	Alkaloids, flavonoids, steroids, proteins, carbohydrates, indigo carmine and essential oils (Linalool and Pinene) were detected in the methanolic extract
Anti-inflammatory property *in vivo* (Chen et al., 2013a)	Eight phenolic compounds were quantified: salicylic acid, syringic acid (major compounds) ρ-coumaric acid, vanillin, syringaldehyde, quercetin, isoliquiritigenin, formononetin. Salicylic acid was found in the plasma of mice fed with *I. suffruticosa* extracts
*In vivo* increase of Phase II detoxification enzyme and glutathione levels (Chen et al., 2013b)	The authors reported the same compounds quantified by Chen et al. (2013a). Ethanolic extracts showed the best action on the induction of phase II detoxification enzyme, and syringic acid was the most active among phenolic compounds detected, however, it was less potent than ethanolic extracts

Synergistic assessments have become a key tool in phytomedicine research in recent years, and uses of antibiotics in combination with herbal products have been investigated as antimicrobials for *S. aureus* resistant strains (Wagner and Ulrich-Merzenich, 2009). Some studies have used erythromycin, a 14-membered ring macrolide antibiotic and therefore part of the Macrolide-Lincosamide-Streptogramin-B (MLSB) family, as a representative drug to evaluate combinatory effects of plant-derived products (Chan et al., 2013, 2015). Antibiotics from the MLSB family serve as an important combatant against *S. aureus* methicillin resistant (MRSA) strains, which are a major cause of disease in the general population and hospital-acquired infections (Pantosti, 2012). MLSB comprises three unrelated groups (macrolide, lincosamide and streptogramin-B) that share the same binding site in bacterial ribosome. It is possible, therefore, that a synergistic effect for one group might predict a similar action from the other groups.

Given this background, our study aimed to define the antimicrobial activities of different organic extracts from *I. suffruticosa* leaves against *S. aureus* strains (MRSA and MSSA), and then to examine synergistic actions with erythromycin.

## Materials and methods

### Chemicals

Dimethylsulfoxide (DMSO), erythromycin and 7-hydroxy-3H-phenoxazin-3-one-10-oxide sodium salt (Resazurin) was purchased from Sigma-Aldrich Chemical Company, St. Louis, MO, while Mueller-Hinton Agar and Nutrient Agar medium were from HIMEDIA Laboratories®. Diethylether, chloroform and acetone were obtained from Merck, Darmstadt, Germany.

### Plant material and preparation of organic extracts

Leaves of *I. suffruticosa* were collected in São Caetano, Pernambuco, Brazil (latitude: 08° 19′ 33″ S; longitude: 36° 04′ 21″ W) between 10 and 11 a.m. The plant was identified by Dr. Marlene Carvalho Alencar Barbosa (Department of Botany, UFPE) and a voucher specimen deposited at the UFP Geraldo Mariz Herbarium-UFPE (identification number 45.217).

Organic extracts were prepared by successively extracting dried leaves of *I. suffruticosa* (100 g) with 200 mL of diethyl ether, chloroform or acetone, common solvents arranged in order of increasing polarity. Briefly, the leaf powder was homogenized firstly with 200 mL of diethyl ether for 2 h in a mechanical stirrer, kept refrigerated overnight (4°C) and filtered with Whatman no.1 paper. The solvent was then removed under reduced pressure in a rotary evaporator at 45°C to produce diethyl ether extract. The plant material which was not extracted by diethyl ether was then homogenized with 200 mL chloroform and all extraction process was repeated generating the chloroform extract. Finally, the remaining powder was submitted to acetone extraction to produce acetone extract. All dried organic extracts of *I. suffruticosa* were stored at −20°C until use and dissolved in dimethyl sulfoxide (DMSO, 1%) before each test.

### Phytochemical screening

An approximate amount of diethyl ether, chloroform and acetone extracts from *I. suffruticosa* leaves were subjected to phytochemical analysis to ascertain the presence of secondary metabolites such as alkaloids, flavonoids, phenylpropanoids, triterpenoids and volatile oil in according to Wagner and Bladt (2009). Briefly, compounds classes were visualized as aid thin layer chromatography (TLC) on silicagel 60 F254 (Merck), mobile phase standard and Dragendorff, NEU-PEG, KOH-Ethanol, Liebermann-Burchard and vanillin-sulfuric acid reagents, respectively. Tests for tannins, saponins and other heterosides were not performed due to the low polarity of the extracts.

### Antimicrobial assays

#### Staphylococcus aureus strains

The antimicrobial activity was tested against the following microorganisms provided by the Departamento de Antibióticos, Universidade Federal de Pernambuco (UFPEDA): *Staphylococcus aureus* (UFPEDA 02), and some isolated strains of *S. aureus* originally obtained from: vaginal secretion (UFPEDA 660); catheter tip (UFPEDA 663); urine sample (UFPEDA 670); blood sample (UFPEDA 672); prostate secretion (UFPEDA 676); wound secretion (UFPEDA 677 and 679); ocular secretion (UFPEDA 687). Strains UFPEDA 670 and 672 are classified as MRSA strains (Table 2). All strains were and maintained in Mueller-Hinton Agar (MHA) and stored at 4°C.

**Table 2:** **Susceptibility to antibiotics of *Staphylococcus aureus* strains^a^**.

**UFPEDA**	**Source**	**Susceptibility to antibiotics**			
		**Oxacillin**	**Cefoxitin**	**Erythromycin**	**Clindamycin**
02	ATCC 6538	S	S	S	S
660	Vaginal secretion	S	S	S	S
663	Catheter tip	S	S	S	S
670^b^	Urine sample	R	R	R	R
672^b^	Blood sample	R	R	R	R
676	Prostate secretion	S	S	S	S
677	Wound secretion	S	R	R	S
679	Wound secretion	S	S	R	S
687	Ocular secretion	S	S	S	S

R, resistant; S, sensitive.

a Data provided by UFPEDA Collection.

b MRSA.

#### Determination of antibacterial activity using the disc diffusion method

The antibacterial activity of the organic extracts of *I. suffruticosa* leaves was determined by the disc diffusion method (de Oliveira et al., 2012). Briefly, all clinically isolated *S. aureus* strains were grown on MHA medium at 37°C for 18 h, suspended in distilled water (approximately 1.5 × 10^8^ CFU/mL) and 100 μL aliquots of bacterial suspension were immediately inoculated in Petri dishes containing MHA medium. Sterile paper discs (6 mm diameter) containing 20 μL organic extracts of *I. suffruticosa* (100 mg/mL) were applied to the agar and the Petri dishes incubated at 37°C for an additional 18 h. Following incubation, the diameter of the inhibition zone (DIZ) of growth was measured, using DMSO as negative control.

#### Effects of temperature and pH on antimicrobial activity

The antimicrobial activity of each *I. suffruticosa* extract against *S. aureus* UFPEDA 02 was determined. Samples were placed in sterile tubes and kept for 30 min at different temperatures (28, 30, 60, and 100°C), or were stored at a different pH for 30 min at 25°C, using 1M NaOH or 1M HCl to adjust the pH range between 3 and 10. The antibacterial activity of treated extracts was tested using the disc diffusion method, as described above.

#### Determination of minimum inhibitory concentration (MIC) and minimum bactericidal concentration (MBC)

The minimal inhibitory concentration (MIC) was determined by a microdilution broth susceptibility assay (Clinical and Laboratory Standards Institute, 2011). Two-fold serial dilutions of the organic extracts of *I. suffruticosa* containing 50–0.20 mg/mL in DMSO were prepared in Mueller-Hinton Broth (MHB; 200 μL) in a 96-well microtiter plate. Bacterial suspensions were prepared from each *S. aureus* strains freshly grown in Mueller-Hinton broth (Merck) (approximately 1.5 × 10^8^ CFU/mL,) and 10 μL of this suspension was added to each well. After incubation at 37°C for 24 h, bacterial growth was recorded using a Resazurin solution (0.01%). MIC was the lowest concentration at which no color change (from purple to pink) was observed. Afterwards, cultures were seeded in MHA medium and incubated for 24 h at 37°C to determine the minimum bactericidal concentration (MBC), which corresponds to the lowest amount of extract that kills *S. aureus*. All experiments were performed in triplicate.

#### Evaluation of combinatory effects of extracts and erythromycin

Combinatory effects between organic extracts of *I. suffruticosa* and erythromycin were assessed using the checkerboard test against the strain UFPEDA 02. Briefly, samples with different proportions of plant extract:drug (final volume: 20 μL) were prepared from stock solutions of each extract (50 mg/mL) and erythromycin (1 mg/mL) and antibacterial activity was tested as described for MIC determination (da Silva et al., 2013). The Fractional Inhibitory Concentration (Σ FIC) was calculated according to the equation:

ΣFIC=(MICE+D/MICE)+(MICD+E/MICD)

MICE+D: minimal inhibitory concentration of extract in combination with erythromycin; MICD+E: minimal inhibitory concentration of erythromycin in combination with extract. Results were considered: synergistic (Σ FIC < 0.5); additive (0.5 < Σ FIC < 1); non-interactive (1 < Σ FIC < 4); or antagonist (Σ FIC > 4) (Vuuren and Viljoen, 2011).

### Statistical analysis

Each experiment was performed in triplicate and results are expressed as the mean ± standard deviation (SD). Statistical analyses were performed by ANOVA and unpaired Student's *t*-test. All analyses were carried out using software StatView, version 4.5, Abacus Concept, Inc, Berkeley, CA. Differences were considered significant at *p* < 0.05. The correlation indices were calculated using the Pearson coefficient (ρ).

## Results

### Phytochemical analysis

TLC analysis revealed the presence of flavonoids, phenylpropanoids, triterpenoids and volatile oils in all three extracts. In most of the tests performed, only quantitative differences were found. Thus, flavonoids, phenylpropanoids and volatile oils predominated in acetone, ether and chloroform extracts, respectively. Alkaloids or nitrogen-containing compounds were detected only in the chloroform extract of *I. suffruticosa* (Table 3).

**Table 3:** **Phytochemical analysis of organic extract from leaves of *Indigofera suffruticosa***.

**Compounds class**	***Indigofera suffruticosa* extracts**		
	**Ether**	**Chloroform**	**Acetone**
Alkaloids	-	+	-
Flavonoids	+	+	++
Phenylpropanoids	++	+	+
Triterpenoids	+	+	+
Volatile oils	+	++	+

(-) absent, (+) weak, (++) strong.

### Antibacterial activity of organic extracts from leaves of *I. suffruticosa*

**Figure 1:**
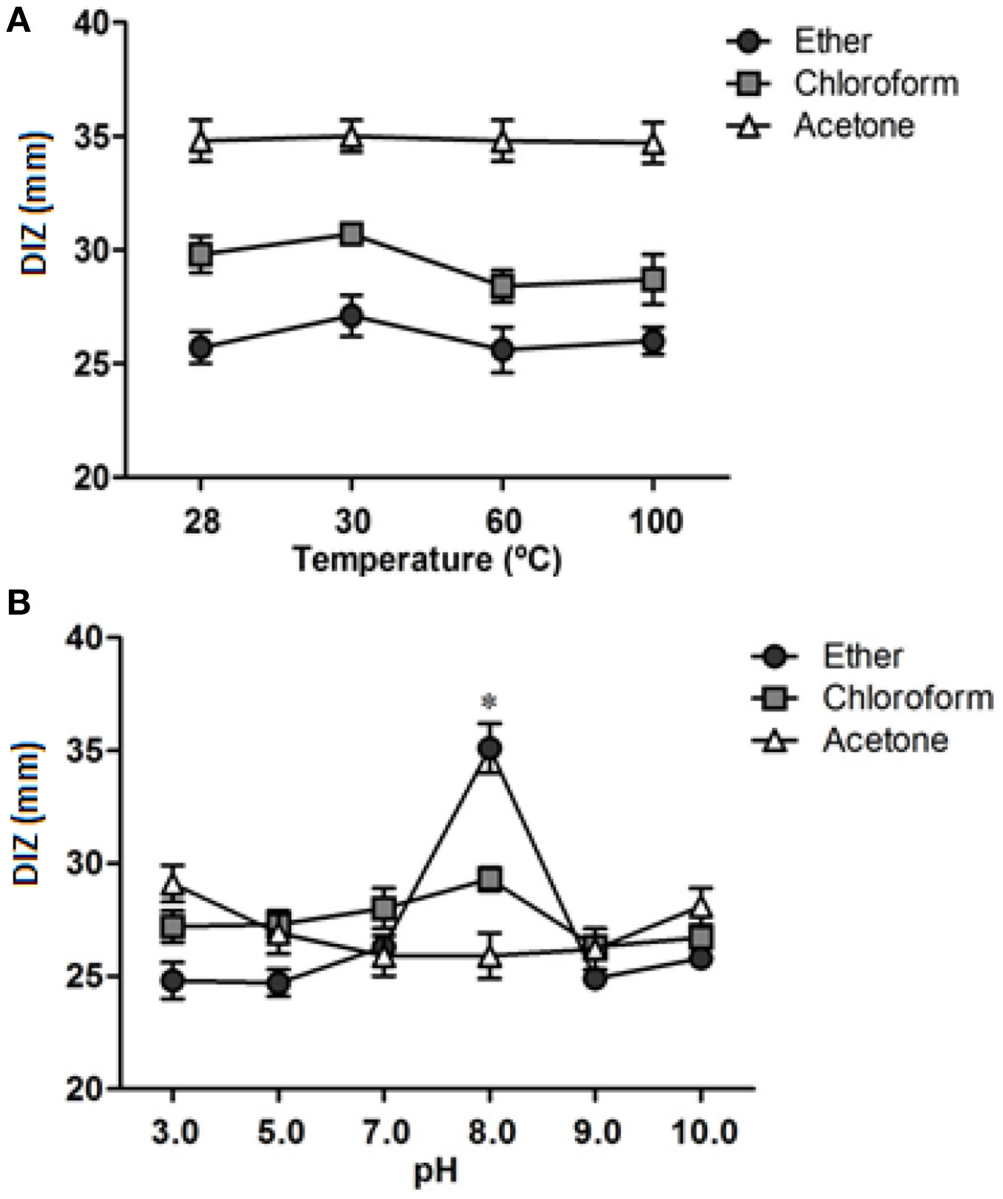
**Stability of organic extracts of leaves of *Indigofera suffruticosa*. (A)** Effect of temperature on the stability of organic extracts of *I. suffruticosa*. **(B)** Effect of pH on the stability of organic extracts of *I. suffruticosa*. DIZ—inhibition zone diameter. ^*^Significant differences in relation to control.

All organic extracts of leaves of *I. suffruticosa* showed antimicrobial activity against different *S. aureus* strains. However, the inhibition varied according to the extract and test strain with DIZ values ranging from 25.3 ± 2.1 to 36.0 ± 1.0 mm (Table 4). All extracts were active against both MRSA strains (UFPEDA 670 and UFPEDA 672) with DIZ values >30.0 mm, except for the chloroform extract which gave a DIZ of 27.7 ± 2.5 mm for strain UFPEDA 670. Diethyl ether extracts showed the best inhibition (30.08 ± 2.69 mm), followed by acetone (28.79 ± 3.35 mm) and chloroform (28.7 ± 3.42 mm), however no significant differences were observed between these average DIZ values (*p* > 0.05). Furthermore, strong correlations were found between the DIZ of all extracts with ρ-values of 0.86, 0.94, and 0.92 for ethyl/chloroform, and chloroform/acetone ethyl/acetone extracts, respectively. The antimicrobial activity of the extracts was not affected (*p* > 0.05) after high temperature treatment (Figure 1A) or variation of pH (Figure 1B), except for the ether extract which was notably more active at pH 8 (*p* > 0.05).

**Table 4:** **Antimicrobial activity of organic extracts from leaves of *Indigofera suffruticosa* against *Staphylococcus aureus* strains**.

***S. aureus* strains**	**Organic extracts of leaves of *Indigofera suffruticosa* DIZ**		
	**Ether**	**Chloroform**	**Acetone**
02	34.7 ± 0.6^a,1^	36.0 ± 0.0^a,1^	35.7 ± 1.1^a,1^
660	29.0 ± 1.7^b,1^	28.0 ± 2.0^b,1^	28.0 ± 2.0^b,1^
663	28.7 ± 0.6^b,1^	27.7 ± 0.6^b,1^	26.7 ± 0.6^b,1^
670	32.7 ± 1.1^a,1^	27.7 ± 2.5^b,2^	30.7 ± 0.6^b,2^
672	32.6 ± 1.1^a,1^	32.3 ± 0.6^c,1^	31.0 ± 3.0^b,1^
676	27.3 ± 0.6^b,1^	25.3 ± 0.6^b,1^	26.3 ± 0.6^b,1^
677	30.0 ± 1.0^b,1^	29.0 ± 1.7^b,1^	29.7 ± 0.6^b,1^
679	29.0 ± 1.0^b,1^	26.3 ± 2.3^b,1^	25.7 ± 2.1^b,1^
687	26.7 ± 2.3^b,1^	26.0 ± 2.6^b,1^	25.3 ± 2.1^b,1^
Average DIZ	30.08 ± 2.7	28.7 ± 3.4	28.78 ± 3.4

DIZ values are expressed in mm.

^*^Same superscript letter (^a,b,c^) indicates no significant difference (p > 0.05) between DIZ values from different strains for each solvent (same column).

^**^Same superscript number (^*1,2*^) indicates no significant difference (p > 0.05) between DIZ values from different solvents against each strain (same row).

The MIC and MBC values ranged from 0.78 to 6.25 mg/mL and 3.12 to 25.0 mg/mL, respectively, with the acetone extract having the lowest values (Table 5). The MIC_50_ (minimum concentration able to inhibit 50% of strains) was 1.56 mg/mL for the acetone extract, and 6.25 mg/ml for both ether and chloroform extracts. Similarly, the MBC_50_ (minimum concentration able to kill 50% of strains), for the acetone extract was 6.25 mg/mL, but 12.5 mg/mL for ether and chloroform extracts. Additionally, the average MIC and MBC of acetone extract (2.16 ± 0.9 and 7.63 ± 3.8, respectively) were lower (*p* > 0.05) than other extracts (4.85 ± 1.6 and 15.27 ± 7.7 for ether extract; and 5.9 ± 1.0 mg/mL and 16.67 ± 6.2 mg/mL for chloroform). The three extracts also differed in their MBC/MIC ratio (Pankey and Sabath, 2004); although ether and chloroform extracts showed exclusively bactericidal effects (MBC/MIC ratios ranged from 2 to 4), the acetone extract had both bactericidal and bacteriostatic actions, however this extract was a bactericidal agent for almost all *S. aureus* strains tested (77.78%).

**Table 5:** **Minimum inhibitory concentration and minimum bactericidal concentration of organic extracts from leaves of *Indigofera suffruticosa* against *Staphylococcus aureus* strains**.

***S. aureus* strains**	**Organic extracts from leaves of *Indigofera suffruticosa***								
	**Ether**			**Chloroform**			**Acetone**		
	**MIC**	**MBC**	**MBC/MIC**	**MIC**	**MBC**	**MBC/MIC**	**MIC**	**MBC**	**MBC/MIC**
02	3.12	12.5	4	3.12	12.5	4	1.56	3.12	2
660	6.25	12.5	2	6.25	25.0	4	1.56	6.25	4
663	6.25	25.0	4	6.25	25.0	4	3.12	12.5	4
670	6.25	25.0	4	6.25	25.0	4	1.56	12.5	8
672	6.25	12.5	2	6.25	12.5	2	3.12	6.25	2
676	6.25	12.5	2	3.12	12.5	4	3.12	3.12	1
677	6.25	25.0	4	3.12	6.25	2	3.12	6.25	2
679	6.25	12.5	2	3.12	6.25	2	1.56	6.25	4
687	6.25	12.5	2	6.25	12.5	2	0.78	12.5	16
MIC_50_	6.25			6.25			1.56		
MBC_50_	12.5			12.5			6.25		
Average MIC	5.9 ± 1.0			4.85 ± 1.6			2.16 ± 0.9		
Average MBC	16.67 ± 6.2			15.27 ± 7.7			7.63 ± 3.8		

MIC, minimal inhibitory concentration; MBC, minimal bactericidal concentration.

MIC_50_, concentration able to inhibit 50% of strains; MBC_50_, concentration able to kill 50% of strains.

MIC, MIC_50_, MBC and MBC_50_ are expressed in mg/mL.

### Combinatory effects of organic extracts of *I. suffruticosa* and erythromycin

When the antimicrobial actions of erythromycin and *I. suffruticosa* organic extracts were tested in combination, additive, synergistic and non-interactive actions were observed (Table 6); importantly, no antagonistic effects were noted. Acetone extract and erythromycin showed synergistic effects (in five ratios (55.56%; Σ FIC values ranged from 0.3 to 0.5), additive effects (0.6 ≤ Σ FIC ≤ 0.8) in three and a non-interactive effect in only one (ratio of 1:9, drug:extract; Σ FIC = 1.7). For the chloroform extract and erythromycin combinations both synergistic (0.2 ≤ Σ FIC ≤ 0.4) and additive (0.7 ≤ Σ FIC ≤ 0.9) effects were equally found in four ratios and only one ratio gave a non-interaction (1:9, drug:extract; Σ FIC = 1.7). No synergistic effect was seen with ether extracts, but 8 ratios resulted in additive effects (0.6 ≤ Σ FIC ≤ 0.9) and 1 ratio a non-interactive effect (3:7, drug:extract; Σ FIC = 1.2). Strong correlations were observed between Σ FIC values from erythromycin/acetone and erythromycin/chloroform combinations (ρ = 0.82), although no significant difference was found between the mean of their Σ FIC values (0.68 ± 0.46 and 0.644 ± 0.44; *p* < 0.05). The best Σ FIC values were 0.2 for erythromycin/chloroform at 5:5, followed by 0.3 for all these combinations: erythromycin/acetone (at 7:3 and 3:7) and for erythromycin/chloroform (at 3:7 and 6:4).

**Table 6:** **Combinatory effects of organic extracts from leaves of *Indigofera suffruticosa* and erythromycin against *S. aureus* UFPEDA 02**.

**Erythromycin/Extracts proportion**	**Organic extracts from *Indigofera suffruticosa* leaves (Σ FIC)**		
	**Ether**	**Chloroform**	**Acetone**
9:1	0.9	0.9	0.4
8:2	0.9	0.4	0.4
7:3	0.7	0.7	0.3
6:4	0.6	0.3	0.6
5:5	0.6	0.2	0.5
4:6	0.8	0.8	0.8
3:7	1.2	0.3	0.3
2:8	0.8	0.8	0.8
1:9	0.8	1.7	1.7
Average Σ FIC	0.81 ± 0.18	0.68 ± 0.46	0.644 ± 0.44

## Discussion

*S. aureus* is a pathogen long-recognized to be capable of developing drug resistance which increases patient treatment time, rate of morbidity and mortality, and associated financial costs (Pantosti, 2012). These factors make the search for new active agents against *S. aureus* highly relevant. In contrast to the well-known antimicrobial effects of *I. suffruticosa* (Leite et al., 2006; Carli et al., 2010), our present work is the first to evaluate organic solvent extracts for activity against clinical isolates of *S. aureus* strains (including two MRSA strains), as well their combinatory effects with a macrolide drug (erythromycin).

The organic extracts from *I. suffruticosa* leaves showed antimicrobial activity against all tested strains of *S. aureus* and, importantly, high inhibition zones were found against MRSA strains (UFPEDA 670 and UFPEDA 672). These two strains were isolated from different sources and exhibited multidrug-resistant profile (oxacillin-cefoxitin-erythromycin-clindamycin). The best anti-*S. aureus* activity was shown by the acetone extract, since its MIC_50_ was 4-fold lower than the MIC_50_ values of the two other extracts. From chemical point of view, the acetone extract contains more flavonoids than ether and chloroform extracts. It is known that different species of genus *Indigofera* including *I. suffruticosa* are rich source of bioactive flavonoids (Hasan et al., 1993; Narender et al., 2006; Varanda et al., 2011; Perez et al., 2013). Previous chemical analysis from *I. suffruticosa* resulted in the identification of four quercetin derivatives. Although our result revealed that the antimicrobial properties might be associated with the presence of flavonoids, a characterization of acetone extract is necessary, even though this has not been our major focus.

We also showed that high temperature (up to 100°C) had negligible effect on the anti-*S. aureus* activity of each extract, which may explain the effective traditional usage of *I. suffruticosa* in infusions prepared by prolonged boiling of its leaves (Corrêa, 1984). Similarly, the antimicrobial activities of our three organic extracts showed little change when submitted to pH values ranging from pH 3 to pH 10. Thermal and pH stabilities are noteworthy factors for development of new antimicrobial formulations by the cosmetic, food and pharmaceutical industries, and our findings encourage further research into use of our organic extracts.

Exploring combinatory effects of antimicrobial agents and natural products is an attractive strategy to overcome bacterial resistance (Betoni et al., 2006; Wink et al., 2012). Diverse targets are involved in the synergistic effects of drugs and plant-derived products such as enzymes and substrates, metabolites, receptors, ion channels, transport proteins, DNA and RNA (Wagner, 2011; Yang et al., 2014). Our study establishes that all organic extracts from *I. suffruticosa* induce at least additive effects with erythromycin. In addition to its more potent antimicrobial activity, the synergestic effect of the acetone extract was higher than that of the chloroform extract, although this did not reach statistical significance and the Σ FIC values of the two were strongly correlated. In contrast, the *I. suffruticosa* ether extract only showed additive effects or, in one tested ratio, a non-interactive effect. These results suggest these as a promising source of potential compounds to be used in combination of erythromycin (and other members of MLSB family).

*I. suffruticosa* extracts have been target of a various studies in order to prove their medicinal potential. Most of these works have shown that polar solvent extracts are more active (Table 1) as they are rich in phenolic compounds, flavonoids, carbohydrates, glycoproteins, indigo, alkaloids, and triterpenes (Leite et al., 2006; Carli et al., 2010; Lopes et al., 2011; Luiz-Ferreira et al., 2011; Almeida et al., 2013; Chen et al., 2013a,b). Furthermore, extracts from *I. suffruticosa* have been also shown key features to be used as a medicine such as lethal dose 50% (1600 mg/kg (ip) in mice (Almeida et al., 2013) and induction of phase II detoxification enzyme and increase of glutathione levels in rat Clone 9 liver cells (Chen et al., 2013b).

In summary this paper showed that organic extracts of *I. suffruticosa* are promising natural products for the development of new anti-*S. aureus* formulation given their antimicrobial inhibiting MRSA strains and their combination with erythromycin seems to be very perspective, thus deserving further studies in order to understand their mechanism of action.

### Conflict of interest statement

The authors declare that the research was conducted in the absence of any commercial or financial relationships that could be construed as a potential conflict of interest.
